# Significant pharmacokinetic differences of berberine are attributable to variations in gut microbiota between Africans and Chinese

**DOI:** 10.1038/srep27671

**Published:** 2016-06-10

**Authors:** Raphael N. Alolga, Yong Fan, Zhuo Chen, Li-Wei Liu, Yi-Jing Zhao, Jin Li, Yan Chen, Mao-De Lai, Ping Li, Lian-Wen Qi

**Affiliations:** 1 State Key Laboratory of Natural Medicines, China Pharmaceutical University, Nanjing, Jiangsu, China; 2 Department of Emergency Center, the First Affiliated Hospital of Nanjing Medical University, Nanjing, Jiangsu, China.

## Abstract

We investigated the influence of gut microbiotal metabolism on the pharmacokinetics of berberine in healthy male Africans and Chinese. The *C*_*max*_ and *AUC* in the Africans were 2.67-fold and 2.0-fold higher than the Chinese, respectively. Microbiotal compositions by 16S rRNA pyrosequencing showed higher abundance of the genera *Prevotella*, *Bacteroides*, and *Megamonas* (34.22, 13.88, and 10.68%, respectively) in the Chinese than the Africans (30.08, 9.43, and 0.48%, respectively). Scatter plot showed a strong negative correlation between the microbiotal abundance and the berberine *AUC*, especially for the genus *Prevotella* (*r* = −0.813) and its species. A more extensive metabolism was observed in Chinese with 1.83-fold higher metabolites, possibly contributing to the lower *AUC* than the Africans. In conclusion, significant PK differences of berberine were observed between Africans and Chinese, which is partly attributable to variations in gut microbiota and its corresponding metabolic capacity.

Berberine is an active constituent present in many medicinal plants. It has long been used in traditional medicine to treat many health concerns, and recently has been widely tested due to its diverse clinical and pharmacological activities^1^,^2^,^3^,^4^,^5^. It has now become one of the world’s most widely used natural products. It is estimated that, approximately 20 billion pills of berberine are consumed every year in Asian countries. Because of its low cost, berberine has also been recognized and has gained entry in many African countries. In USA, it is widely marketed as a powerful dietary supplement.

Pharmacokinetic (PK) profile of berberine is necessary data to design a rational dosage regimen. Berberine has low rates of absorption when taken orally. High oral doses may cause intestinal side-effects, including constipation, stomach upset, and cramping^4^. For this reason, choosing the right dose of berberine for a specific population is of significance to avoid potential side-effects. Though berberine is largely used in many African countries, PK studies of the drug in such races are lacking. Most African countries are bedeviled with myriad of problems and with little to no technological know-how to conduct their independent PK studies. They are usually left with no option than follow the dosage regimen gotten from other races.

PK differences in response to drugs have been of concern in different racial populations and ethnic groups. Pharmaceutical companies do include specific genetic or racial information to some marketed drugs to guide their usage by various populations. Isosorbide dinitrate-hydralazine is known to be effective for use in times of heart failure in black patients^6^. Warfarin and rosuvastatin are required in a lower dose by Asians^7^,^8^, while tacrolimus is needed at a higher dose by blacks^9^. Some of the observed racial differences may be explained by the genetic differences^10^. Other possible mechanisms for the differences remain unknown. Understanding these differences is of clinical significance for individualized treatments.

The role of the gut microbiota in humans is under scientific scrutiny and receiving a lot of attention from the scientific community^11^,^12^,^13^. It is referred as a “hidden organ” of the body. Several factors have been found to participate in shaping the gut microbiota. These include, mode of birth, age, diet, race, the environment, diseases, and antibiotic use to mention a few^14^,^15^,^16^. The importance of the gut microbiota in the PK of drugs cannot be gainsaid. In many cases, inter-individual and intra-individual differences in drug metabolism could be linked to variations in the gut microbiota^17^. Gut microbiota might be the key phase responsible for the blood/systemic absorption and circulation of drugs^18^.

In this work, we used a combination of mass spectrometric, microbiomic, and multivariate statistical tools to assess PK differences of berberine between young healthy male African and Chinese volunteers, and to investigate the relationship of gut microbiota with blood concentration.

## Results

### Pharmacokinetic Differences between Africans and Chinese

**Figure 1:**
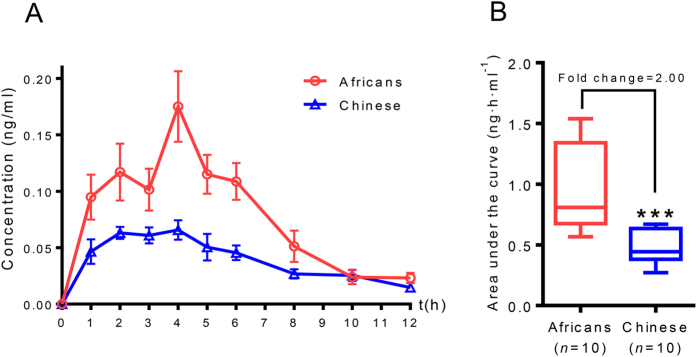
Plasma concentration-time curves and areas under the curve. Results are shown for the plasma concentration-time curve of berberine (Panel A; values are represented as means ± SEM). Panel B shows the box plot of areas under the curve in 12 hours for 10 Africans and 10 Chinese (***represents difference with a *p* value < 0.001). *P* values are analyzed based on student’s *t*-test.

The PK profile for the volunteers showed that there were significant differences between the Chinese and Africans as summarized in Table 1. The *C*_*max*_ were respectively 0.16 ± 0.08 and 0.06 ± 0.02 ng·mL^−1^ for the Africans and Chinese (*p* = 0.0006). This represents an increase of 2.67-fold. Significantly higher area under the curve (*AUC*) values were observed in the Africans (0.96 ± 0.34 ng·h·mL^−1^) compared with the Chinese subjects (0.47 ± 0.13 ng·h·mL^−1^) with a *p* value of 0.0037, indicating a 2.0-fold increase in the Africans (Fig. 1A,B). There was no significant difference in *t*_*max*_ between the two races, 4.00 ± 1.38 h and 4.00 ± 1.34 h for Africans and Chinese, respectively. Significant differences were observed for apparent volume of distribution (*p* = 0.0125), and total body clearance (*p* = 0.0201) in Africans and Chinese.

### Variations in Gut Microbiota Composition

**Figure 2:**
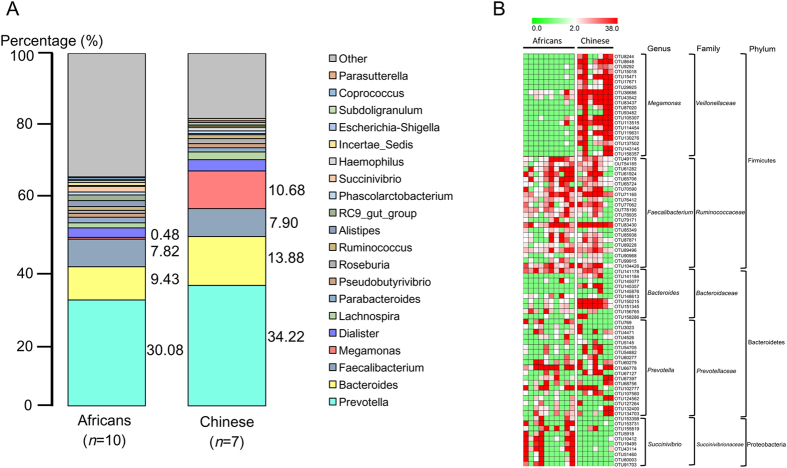
Intestinal microbiota communities of Chinese and Africans. (**A**) The relative abundance map for the bacteria in the stool were analyzed by 16S rRNA pyrosequencing. Feces were collected from all the participants enrolled in the pharmacokinetic study. The values were represented as the average abundance of each genus in the metagenomics test (percentage). (**B**) Heatmap and represented bacterial taxa information (species, genus, family and phylum) of 81 significantly changed OTUs between Africans and Chinese.

Microbiota compositions of fecal samples were determined by 16S rRNA pyrosequencing. Figure 2A is the comparative presentation of the bacterial taxonomic analysis of intestinal microbiota between Africans and Chinese. Figure 2B is the heatmap showing the abundance of 81 significantly changed OTUs between the Africans and Chinese and their representative bacterial taxa information. Gut microbiota compositions showed significant variations. Of the 20 major bacteria genera identified (Fig. 2A), the predominant phyla in both races were *Bacteroidetes* and *Firmicutes.* The genera of most of the bacteria identified from the intestinal microbiome of the Chinese were more abundant than those from the Africans. The predominant genera from the phylum *Bacteroidetes* were *Prevotella* (30.08% for Africans and 34.22% for Chinese) and *Bacteroides* (9.43% for Africans and 13.88% for Chinese). *Faecalibacterium* (7.82% for Africans and 7.90% for Chinese), *Dialister* (2.67% for Africans and 3.15% for Chinese) and *Lachnospira* (1.45% for Africans and 2.20% for Chinese) were the dominant genera from the phylum *Firmicutes* in both races. There was a sharp distinction between the two races in bacteria from the genus *Megamonas*, where the percentage abundance was 0.48% and 10.68% for Africans and Chinese respectively. Bacteria from two genera, *RC9* gut group (1.63% abundance) and *Succinivibrio* (1.56% abundance) were exclusively detected in the intestinal microbiome of Africans. The heatmap presentation in Fig. 2B gives a much clearer picture of the significant differences observed in terms of the bacteria population distribution between the two races. The change in color intensity from green towards red indicates the relative abundance of the group of bacteria concerned. In concordance with our observation, the heatmap confirmed the major difference between the Africans and Chinese to be from the genera of *Prevotella, Bacteroides*, *Faecalibacterium, Megamonas,* and *Succinovibrio*.

### Correlation Analyses between Bacteria Abundance and AUC of Berberine in Participants

**Figure 3:**
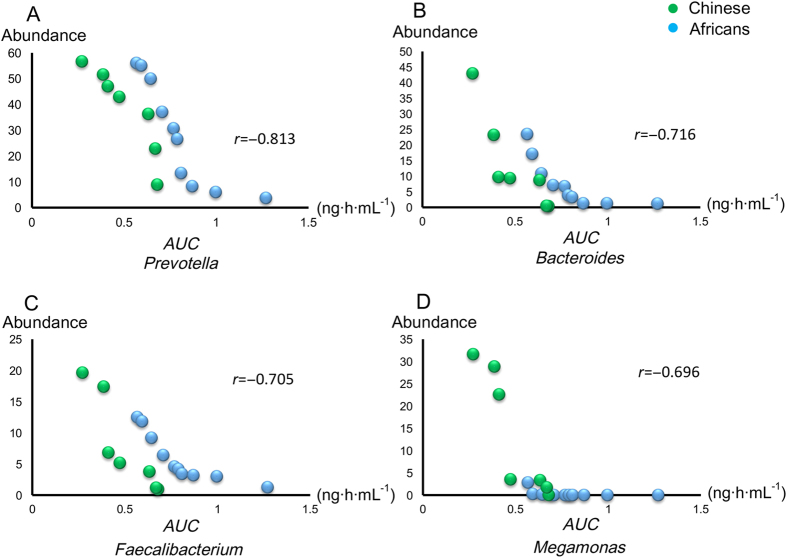
The correlation between areas under the curve (*AUC*s) and abundances of intestinal genus *Prevotella* (**A**), *Bacteroides* (**B**), *Faecalibacterium* (**C**), and *Megamonas* (**D**). Green plots represent Chinese participants, and blue plots represent African volunteers.

The relationship of gut microbiota with the PK was plotted in a network by correlating the bacterial OTUs with the *AUCs* of berberine in participants. As shown in Supplementary Fig. 1, 34 OTUs and 21 OTUs screened from a total of 360 OTUs displayed a highly negative correlation (*r* < −0.7) with the individual *AUC* for the Africans and Chinese, respectively. These highly correlated OTUs belong to *Prevotella*, *Bacteroides, Faecalibacterium,* and *Megamonas*. There were 9 shared OTUs for the Africans and Chinese and found to belong to the genera of *Bacteriodes* (2 OTUs) and *Prevotella* (7 OTUs). Figure 3 presents a holistic representation of these bacteria genera in all the participants in relation to their link with the *AUC* of berberine. The higher the correlation, the greater the contribution of the particular genus towards the plasma concentration of berberine. Bacteria from the genus *Prevotella* showed the highest correlation with *AUC* (*r* = −0.813) while those belonging to *Megamonas* were least correlated with *AUC* (*r* = −0.696) in both the African and Chinese groups. The negative correlation values signify the existing inverse relationship of the bacterial abundance with the *AUC* of berberine. The contributions of these genera towards the metabolism of berberine, hence, its *AUC* in all participants from the results is follows: *Prevotella* > *Bacteriodes* > *Faecalibacterium* > *Megamonas*.

### Gut Microbiotal Metabolism of Berberine

**Figure 4:**
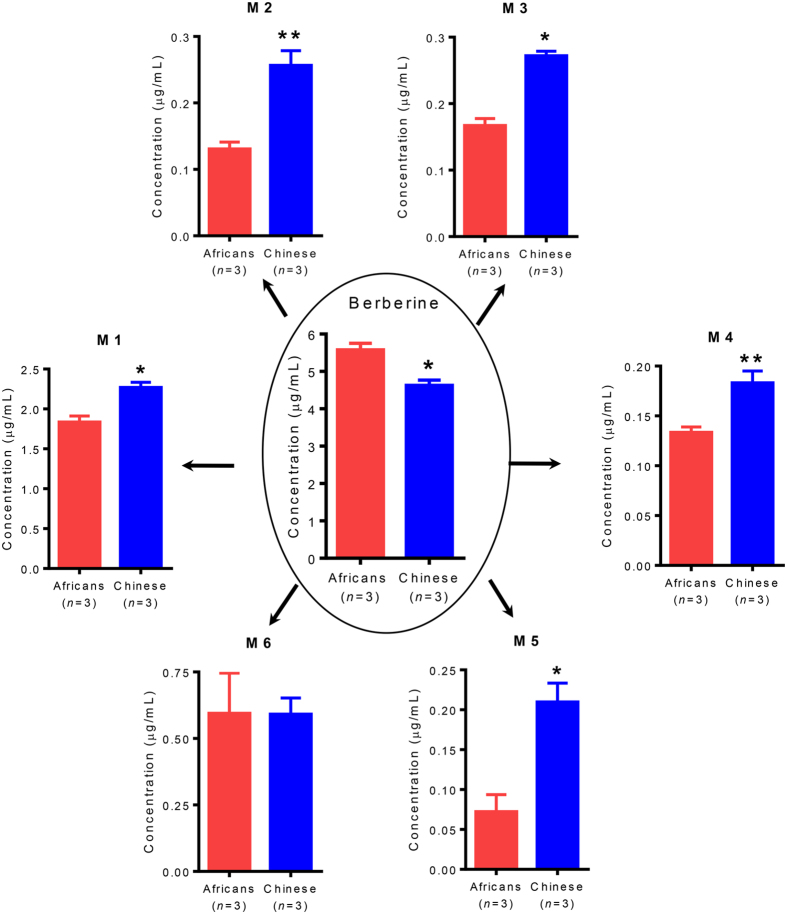
Intestinal metabolism of berberine. Results are shown for the concentrations of berberine left and 6 major metabolites generated in the medium after anaerobic incubation with intestinal microbiota for 48 hours at a concentration of 1 μmol/L *in vitro.* The intestinal microbiota was randomly collected from 3 Africans and 3 Chinese participated in the pharmacokinetic analysis. Concentrations were represented as means ± SEM. The concentrations of dihydroberberine (M6) were observed to be equal between races. *p value < 0.05; and **p value < 0.01. *P* values are based on Student’s *t*-test analyses. M1, Berberrubine; M2, Thalifendine; M3, Columbamine; M4, Demethyleneberberine; M5, Jatrorrhizine; M6, Dihydroberberine.

To confirm the microbiota-PK connectivity, microbiotal metabolic capacities in the two races were investigated *via* anaerobic incubation of berberine with fecal microbiota from the participants. Six major metabolites were detected as shown in Fig. 4. The amount of berberine remaining after incubation for 48 hours was significantly different between the Africans and Chinese (*p* = 0.0126). The left-over berberine was higher in the culture media obtained from Africans than the Chinese. The most abundant metabolite was berberrubine (M1). Other moderately high metabolites included thalifendine (M2), columbamine (M3), demethyleneberberine (M4), jatrorrhizine (M5), and dihydroberberine (M6). Compared with the Africans, a more extensive metabolism was observed in Chinese with 1.83-fold (95% confidence interval, 1.02–2.64) higher metabolites, including M1 (*p* = 0.0118), M2 (*p* = 0.0067), M3 (*p* = 0.0011), M4 (*p* = 0.0014), and M5 (*p* = 0.0122). The only exception was with M6 which was found to be almost equal between the two races (*p* = 0.9846).

## Discussion

Berberine is used in many African countries at the same dosages as that used by the Chinese. Recommending the suitable dose of berberine for a particular racial group is of importance to allow a certain degree of personalized therapy and reduction in adverse drug reactions. In this study, to our surprise, significant differences were observed in *C*_*max*_ and *AUC* between the Africans and Chinese. The *C*_*max*_ for the Africans was higher than that of the Chinese by a factor of 2.67. The *AUC* was 2.0-fold higher in the Africans than in the Chinese. The time taken to reach the maximum plasma concentrations (*t*_*max*_), however, was almost the same for the two races, 4 ± 1.38 h and 4 ± 1.34 h for Africans and Chinese respectively. These findings indicated that with the same dosage of berberine, distinct maximum plasma concentrations would be reached at almost the same time by young male Africans and Chinese. This observation further points to the fact these two races should not take the same dose of berberine if adverse effects are to be avoided, and efficacy remains prime. The difference in the *AUC* of 2.0 suggests that the required effective and safe dose for the young male Africans, is half that of the young male Chinese.

The mechanism behind the racial PK differences was then investigated. We proposed a hypothesis that gut microbiota played a key role in determining the plasma concentrations of berberine. We tested the human fecal microbiotal compositions (as a proxy for gut microbiota) for the volunteers by 16S rRNA pyrosequencing. Compared with the Africans, Chinese showed a higher abundance of the main bacterial genera, *i.e*., *Prevotella*, *Bacteroides*, and *Megamonas*. The high prevalence of *Bacteriodetes* and *Firmicutes* phyla of bacteria suggests a healthy normal gut microbiota. Murphy *et al.* reported that *Bacteroides* and *Prevotella* are negatively correlated with energy intake^19^. The relatively lower levels of these two genera in Africans could mean that the Africans have a higher energy intake than the Chinese. These genera are also associated with the production of short-chain fatty acids (SCFAs). Additionally, the presence of the *Succinivibrio* bacteria in only Africans relates to the consumption of cereals and grains. This genus has been reported to be in the gut of animals that feed on grains^20^. Another genus, RC9 gut group exclusively found in the Africans has also been detected in the gut of non-ruminant herbivores and believed to be involved in the metabolism of non-digestible fibre^21^.

Although the importance of the gut microbiome in drug response is now widely recognized, it is not yet known which species out of the many are of ‘‘key’’ importance in influencing blood concentration of berberine. The potential correlations were analyzed between human fecal microbiota and the blood concentration of berberine. A general negative correlation was observed between the bacteria OTUs and the *AUC* of berberine, which reflects the fact that the concentration of berberine in the plasma is inversely related to the bacteria OTU. The lower the concentration of berberine is, and the greater the bacterial populations are. The bacterial OTUs highly correlated with *AUC* were from four main genera, *Prevotella, Bacteroides*, *Faecalibacterium*, and *Megamonas*. Of all these highly correlated bacterial OTUs with *AUC*, nine OTUs were common between Africans and Chinese. The bacteria to which these OTUs belong are from the *Bacteroides* and *Prevotella* genera. Further analysis of the correlation of these four genera with *AUC* suggests the possible contributions of each genus towards the berberine PK. Scatter plot showed a strong negative correlation between the microbiotal abundance and the berberine *AUC*, especially for the genus *Prevotella* (*r* = −0.813).

The gastrointestinal microbiota can be considered as a site with huge biotransformational capacity of drugs^22^,^23^,^24^,^25^. In many cases, inter- and intra-racial differences in drug PK could be linked to variations in the gut microbiotal metabolism. To confirm the microbiota-PK connectivity, microbiotal metabolic capacities in different races were investigated. Consistent with our hypothesis, using fecal microbiota to incubate berberine, a more extensive metabolism was observed in Chinese. We also tried to detect the metabolites in blood, and observed that their concentrations were much lower than that of the berberine, indicating that this drug is absorbed into the blood as the parent compound. The higher abundances of *Prevotella*, *Bacteroides*, and *Megamonas* responsible for the metabolism of berberine account for better metabolic capacities and thus higher metabolites in the Chinese gut. This invariably determines the downstream effects on lower blood concentration of the parent drug in the Chinese.

The clinical significance of these differences is with the dosage adjustment. Thousands of people in Africa are consuming berberine, and many Africans living in Asia are also potential berberine consumers. Based on our PK data, an average African male will need to take about 100 mg instead of 200 mg taken by a Chinese. Enhanced absorption observed in Africans can reduce the dose of berberine required to achieve therapeutically equivalent effects in Chinese population.

Our study has some limitations. This study relates to the gut microbiotal influence in two racial populations, Africans and Chinese. Further research is recommended with a larger ethnic and culturally diverse population. Our study group consisted of young and healthy adults. We also recommend that patients with metabolic disorders such as diabetes could be recruited for a comparative evaluation.

In conclusion, our results have shown significant differences in the blood absorption and PK characteristics of berberine between African and Chinese. These differences depend largely on the variations in gut microbiota, and partly due to the corresponding intestinal metabolic capacities. It should be noted that many other drugs when given orally might have similar differences among various races and even in the same country with ethnic diversity. An effective and safe dosage regimen of these drugs should be designed carefully in clinical prescription, especially for those with narrow therapeutic windows. Microbiotal compositions should be considered important in personalized dosing strategies.

## Methods

### Participants

The study involved a total of 20 non-smoker male volunteers (*i.e.* 10 Africans and 10 Chinese). These were randomly recruited on mutual consent and after each provided a written informed consent of participation. The mean age (y) and BMI (kg/m^2^) (±SD) were 23.35 ± 5.40 and 22.40 ± 2.30 respectively for the Africans, while that for the Chinese were 24.40 ± 3.50 and 22.30 ± 3.70 for the mean age and BMI respectively. All participants were declared medically fit with acceptable physical and medical histories such as complete blood counts, platelets, urinalysis, electrocardiogram, Chest X-ray, electrolyte tests, liver and kidney function tests. Persons with histories of recurrent diarrhea, diabetes, obesity and gastrointestinal disorders were excluded from the study. Those who also had taken any antibiotic in the previous two months were exempted. Finally berberine-containing food or medications were avoided at least 21 days prior to the study. The details of participants are shown in Supplementary Table 1. The study was approved by the Ethics Committee of the First Affiliated Hospital of Nanjing Medical University (2015-SRFA-085) and conducted under the guidelines of the Helsinki Declaration and the International Conference on Harmonization-Good Clinical Practices (ICH-GCP).

### Pharmacokinetic Study

The study was conducted under the guidelines of the Helsinki Declaration and the International Conference on Harmonization-Good Clinical Practices (ICH-GCP). All participants fasted 12 hours prior to the study. Each participant took a single dose of 600 mg of berberine hydrochloride (net dose of 542.8 mg) and a standard breakfast of two slices of unleavened bread and a bottle of water. Blood samples were taken by a phlebotomist at the following time points: 0 h (*i.e.* before ingesting the drug), 1, 2, 3, 4, 5, 6, 8 and 12 h (after taking the drug) into heparinized tubes. The blood samples were centrifuged and the plasma was stored at −80 °C for analysis. Standard meals were provided for lunch and dinner.

### Sample Preparation

An aliquot of 450 μL of acetonitrile was added to 150 μL of each plasma sample and the mixture vortexed for 10 seconds. At 4 °C, the mixture was centrifuged for 10 minutes at the speed of 13,000 rpm. 450 μL of supernatant from each sample was transferred to clean tubes and dried under a gentle stream of nitrogen at room temperature. Finally, the dried supernatant was each dissolved with 80 μL water/acetonitrile (4:1) solution containing tetrahydropalmatine as the internal standard at a concentration of 50 ng/mL.

### Instrumental Analysis

The prepared plasma samples were analyzed using Agilent 1290 infinity system (Agilent Corp., Santa Clara, CA, USA). Separations were done using Agilent Eclipse (100 mm × 2.1 mm, 1.7 μm) C18 column at 50 °C. The mobile phase consisted of aqueous 0.1% formic acid solution (A) and acetonitrile with 0.1% formic acid (B). The column was eluted in a linear gradient with 1–20% B over 0–1 min, 20–70% B over 1–3 min, 70–85% B over 3–8 min, 85–100% B over 8–9 min, the composition was held at 100% B for 1 min. The flow rate and injection volume were respectively 0.4 mL/min and 1 μL. Detections of the separation was done by Agilent Q/TOF mass spectrometer (Agilent Corp., Santa Clara, CA, USA) equipped with an ESI interface. The operation parameters were as follows: drying gas N2 flow rate, 10.0 L/min; temperature, 330 °C; nebulizer, 35 psig; capillary, 3500 V; fragmentor voltage 120 V. The samples were analyzed in the positive ion mode in full scan (50–1000 Da) due its sensitivity for the detection of berberine.

### Method Validation

The developed analytical method was validated according to the FDA guidelines in terms of selectivity, carry-over effect, linearity, accuracy, precision, matrix effect, recovery and LLOQ.

### Fecal Sample Treatment

Three grams of fresh feces from each participant was weighed into a beaker and suspended in 30 mL of normal saline to form slurry. This slurry was then filtered through gauze and centrifuged at 2000 rpm for 30 minutes. The clear supernatant solution was then used for anaerobic incubation with berberine.

### DNA extraction and 16S rRNA Pyrosequencing

Stool samples were freshly collected from all the participants and stored at −20 °C before use. Genomic DNA extraction for each fecal sample from each participant was done using TIANamp Stool DNA Kit according to manufacturer’s protocol. The 16S ribosomal RNA (rRNA) gene was analyzed to evaluate the bacterial diversity by using Illumina Miseq (Novogene Bioinformatics Technology Co., Ltd.).

### Anaerobic Incubation and Detection of Metabolites

One milliliter (1 mL) of aqueous berberine of concentration 0.01 mg/mL and 2 mL of intestinal flora were added to conical flasks containing 30 mL of anaerobic physiological media. The flasks were anaerobically incubated at 37 °C for 48 hours in Whitley DG 250 Anaerobic Workstation (US). The mixtures were then extracted 3 times with 50 mL ethyl acetate. The remaining mixtures were re-extracted 3 times with 50 mL *n*-butanol. The combined n-butanol extracts were then washed 3 times with water. Both extracts were concentrated in vacuo and then diluted to the desired volume with methanol. The ethyl acetate and *n*-butanol extracted contents were mixed and diluted to 1 mL with methanol. These final mixtures were then centrifuged at 13000 rpm for 10 min before injected for UPLC-Q/TOF analysis.

### Bioinformatic analysis of Sequencing Data

Pyrosequencing paired end reads were assigned, truncated and merged using Pandaseq software (V 2.7) to generate raw tags. High quality clean tags from the raw tags were obtained and compared with reference database to detect and remove chimeric sequences using Usearch (V 8.0) to obtain effective tags. QIIME (V1.7) platform was used to analyse the effective tags, and tags with ≥97% similarity were assigned to the same operational taxonomic unit (OTU). The Silva111 16S rRNA database was used based on RDP classifier algorithm to annotate taxonomic information for each OTU sequence. Analysis of alpha diversity; community diversity (Shannon, Simpson), Community richness (Chao1 and Ace), Sequencing depth (Good’s coverage) and Observed species; and beta diversity on both weighted and unweighted unifrac distance metrics of OTUs were calculated with QIIME (Version 1.7.0). Cluster analysis of the unweighted and weighted unifrac distance metrics of OTUs was preceded by principal component analysis (PCA) and Principal coordinate analysis (PCoA). Finally, to interpret the distance matrix, Unweighted Pair-group Method with Arithmetic Means (UPGMA) clustering was employed by QIIME software (Version 1.7.0).

### Data Analysis

The PK data analysis was conducted using PKSolver, a menu-driven add-in Microsoft excel software. The data were evaluated using the non-compartmental, model- independent approach. PKSolver is fast, user friendly and results are generated in Microsoft Word in the form of an integrated report. GraphPad Prism 6 was used for the graphical presentation of the results from the intestinal microbiotal metabolism. A student’s *t*-test was used to compare differences in the PK parameters as well as differences in metabolites from the microbiotal metabolism. *P*-values less than 0.05, 0.01 and 0.001 were considered statistically significant, very significant and highly significant respectively. Pearson correlation test was performed using SPSS package (V 19.0). The Cytoscape software package was used to construct the correlation network.

## Additional Information

**How to cite this article**: Alolga, R. N. *et al.* Significant pharmacokinetic differences of berberine are attributable to variations in gut microbiota between Africans and Chinese. *Sci. Rep.* **6**, 27671; doi: 10.1038/srep27671 (2016).

## Supplementary Material

Supplementary Information

## Figures and Tables

**Table 1:** Pharmacokinetic Parameters of Berberine in African and Chinese Volunteers^1^.

Parameter	Unit	Africans	Chinese	*P* value
*t*_1/2_^2^	h	2.57 ± 1.03	3.95 ± 7.73	0.2681
*t*_max_^3^	h	4.00 ± 1.38	4.00 ± 1.34	0.1707
*C*_max_^4^	ng · mL^−^^1^	0.16 ± 0.08	0.06 ± 0.02	0.0006
Area under the curve_(0–12 h)_	ng · h · mL^−1^	0.96 ± 0.34	0.47 ± 0.13	0.0037
Mean residence time	h	5.74 ± 1.41	6.95 ± 1.20	0.2824
Apparent volume of distribution	mL	1926.46 ± 10.73	5544.41 ± 22.59	0.0125
Total body clearance	mL · h^−1^	517.76 ± 18.55	973.16 ± 48.8	0.0201

^1^Plus–minus values are observed means ± SD.

^2^The time required to reduce the maximum plasma concentration of berberine to a half.

^3^The time at the maximum plasma concentration of berberine.

^4^The maximum plasma concentration of berberine.
