# Supplementary material for: Significant pharmacokinetic differences of berberine are attributable to variations in gut microbiota between Africans and Chinese
Source: Sci Rep. 2016 Jun 10;6:27671. doi: 10.1038/srep27671 (PMC4901288; doi:10.1038/srep27671)
Supplement: Supplementary Information [file srep27671-s1.pdf]

Significant pharmacokinetic differences of berberine are partly attributable to variations in gut microbiota between Africans and Chinese

Raphael N. Alolga[1], Yong Fan[1], Zhuo Chen[1], Li-Wei Liu[1], Yi-Jing Zhao[1], Jin Li[1], Yan Chen[2], Mao-De Lai[1], Ping Li[1,*], & Lian-Wen Qi[1,*]

[1]State Key Laboratory of Natural Medicines, China Pharmaceutical University, Nanjing, Jiangsu; [2]Department of Emergency Center, the First Affiliated Hospital of Nanjing Medical University, Nanjing, Jiangsu

*Correspondence should be addressed to: L.-W.Q (Qilw@cpu.edu.cn) or P.L. (liping2004@126.com).

Conflict of interest/Disclosure: All the authors declare no conflict of interest.

Author contributions: R.N.A. and Y.F. contributed equally to this work; L.-W.Q, P.L., and M.-D.L. designed the research; R.N.A., Y.F., Z.C., L.-W.L., Y.-J.Z., J.L., and Y.C. performed the experiments; R.N.A. and Y.F. analyzed data; and R.N.A., Y.F., and L.-W.Q. wrote the paper.

Supplemetary Figure S1

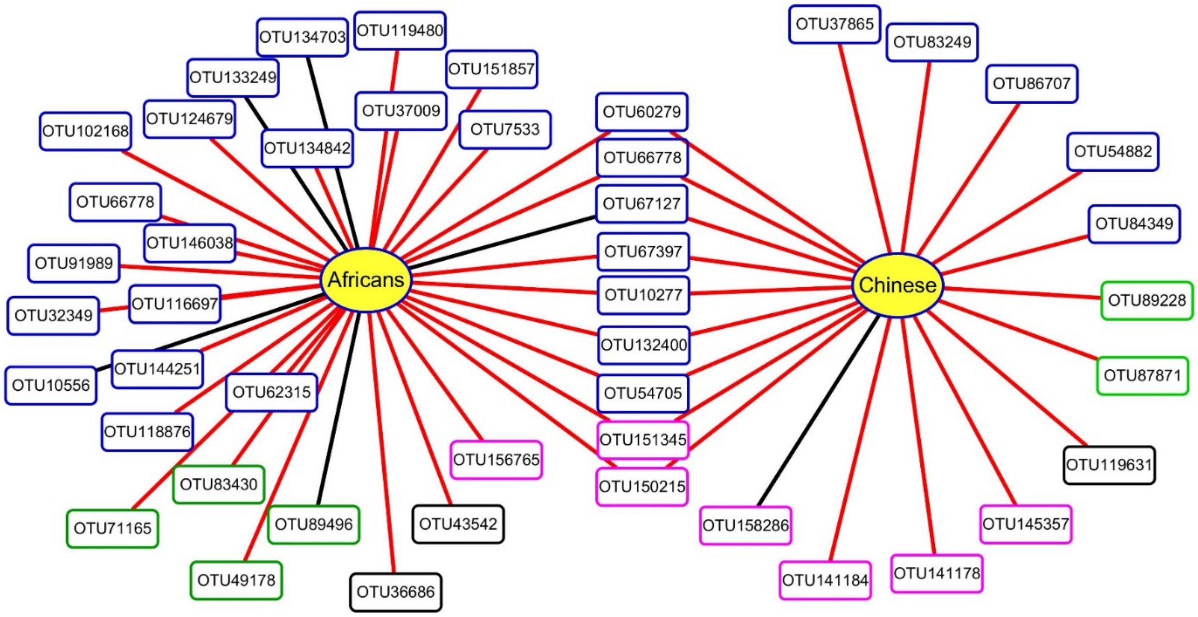

Figure 1. Correlation network of AUC of berberine and bacterial OTUs. Correlation networks of areas under the curve (*AUC*s) and intestinal operative taxonomy units (OTUs) in Chinese and Africans. Highly correlated *AUC*s and OTUs ($r < -0.7$) are connected with a line. OTUs in blue hollow boxes belong to *Prevotella*, OTUs in purple hollow boxes belong to *Bacteroides,* OTUs in green hollow boxes belong to *Faecalibacterium,* and OTUs in black hollow boxes belong to *Megamonas.* Red lines represent negative correlations, and black lines represent positive correlations.

Supplementary Table S1 Volunteer Information.[1]

Participant	Age, y	Body Mass Index[2], Kg/m^2	Country
**African group (*n*=10)**	23.5±5.4	22.4±2.3	
Volunteer 1	21	26.0	Nigeria
Volunteer 2	25	19.3	Uganda
Volunteer 3	20	24.2	Congo
Volunteer 4	25	23.8	Cameroon
Volunteer 5	20	19.7	Zambia
Volunteer 6	20	22.0	Sierra Leone
Volunteer 7	25	20.7	Sierra Leone
Volunteer 8	19	23.0	Nigeria
Volunteer 9	20	22.0	Rwanda
Volunteer 10	37	24.5	DR Congo
**Chinese group (*n*=10)**	24.4±3.5	22.3±3.7	
Volunteer 1	23	31.2	China
Volunteer 2	35	20.7	China
Volunteer 3	25	23.0	China
Volunteer 4	25	20.7	China
Volunteer 5	25	21.9	China
Volunteer 6	23	28.2	China
Volunteer 7	23	17.3	China
Volunteer 8	22	20.0	China
Volunteer 9	20	20.8	China
Volunteer 10	24	19.3	China
***P* value**	0.639	0.975	

[1] Plus–minus values are observed means ± SD.

[2] The body-mass index is the weight in kilograms divided by the square of the height in meters.
